# Revised Orphanet nomenclature and classification for spina bifida and other spinal dysraphisms (SBoD)

**DOI:** 10.1186/s13023-025-03856-4

**Published:** 2025-07-08

**Authors:** Ferdinand Dhombres, Timothée de Saint-Denis, Dominic Thompson, Julie Tahraoui-Bories, Caterina Lucano, Ana Rath, Giovanni Mosiello, Jean-Marie Jouannic, Rennes Workshop #1 attendees, Iris Arsenakis, Maria Paola Bonasoni, Charlène Brochard, Diletta Bruno, Leonardo Caforio, Valeria Capra, Elena Carreras, Matei Claudiu, Darach Crimmins, Sandrine Denoual, Christele Dubourg, Valérie Dupé, Giacomo Esposito, Isabella Fabietti, Marie Faoucher, Wout Feitz, Benoit Fourcroy, Romy Gander, Tamara Geppert, Carlos Giné, Mathilde Gouesse, Lucie Guilbaud, Jacques Kerdraon, Mark Koen, Kolja Kvist, Jane Leonard, Manuel Lopez, Nerea Maiz Elizaran, Rafik Mansouri, Andréa Manunta, Michal Maternik, Luca Mazzone, Mar Melendez, Dario Guido Minoli, Giovanni Montini, Tobias Nientiedt, Sylvie Odent, Matthieu Peycelon, Benoit Peyronnet, Carlota Rodo Rodriguez, Sylvia Roozen, Gloria Fatou Royo Gomes, Roumaisah Saidi, Martin Salö, Emmanuelle Samson, Ammi Sundqvist, Ellen Vandamme, Alain Verloes, Roma Workshop #2 attendees, Eleonore Blondiaux, Elena Carreras, Enrico Castelli, Gessica Dellabella, Jan Deprest, Roland Devilieger, Giacomo Esposito, Lucie Guilbaud, Nerea Maiz Elizaran, Barbara Daniela Iacobelli, Carloefisio Marrass, Luca Massimi, Luca Mazzone, Nicole Ochsenbein, Paolo Palma, Agnieszka Pastuszka, Claudia Rendeli, Dominic Thompson, Barcelona Workshop #3 attendees, Kate Abrahmson, Petra Aden, Papatya Alkan, Giulia Blasetti, Elena Carreras, Gessica Dellabella, Michaela Dellenmark Bloom, Jan Deprest, Giacomo Esposito, Benoit Fourcroy, Carlos Giné, Lucie Guilbaud, Mirthe Klein Haneveld, Manuel Lopez, Nerea Maiz Elizaran, Mar Meléndez, Ueli Moehrlen, Rien Nijman, Agnieszka Pastuszka, Christian Radmayr, Carlota Rodo Rodriguez, Sylvia Roozen, Gloria Royo, Federico Scorletti, Ammi Sundqvist-Andersson, Dominic Thompson, Élida Vazquez, Alain Verloes, ERN network managers, Anne Hugon, Michelle Battye

**Affiliations:** 1 Fetal Medicine Department, Armand Trousseau Hospital, APHP Sorbonne University, GRC 26 and INSERM Limics, Paris, France; 2 Pediatric Neuro-Orthopedic Department, Armand Trousseau Hospital, APHP Sorbonne University, Paris, France; 3 Department of Paediatric Neurosurgery, Great Ormond Street Hospital, London, UK; 4 INSERM, US14 - Orphanet, Plateforme Maladies Rares, 75014 Paris, France; 5 Division of Neuro-Urology, Bambino Gesù Children’s Hospital, IRCCS, 00165 Rome, Italy

**Keywords:** Spinal dysraphism, Rare disease, Classification, Spina bifida, Myelomeningocele

## Abstract

**Background:**

The Spina Bifida and other Dysraphisms working group (SBoD WG) is an interdisciplinary group, comprising experts on spinal dysraphism from 11 European countries. In 2022, the SBoD WG was tasked by 2 European Rare Disease Networks (ERN ITHACA and ERN eUROGEN) to revise the Orphanet classification of spinal dysraphism. Over the past two decades numerous subcategories of spinal dysraphism have been described in the medical literature resulting in a proliferation of terms, numerous synonyms and variously applied definitions. In the light of this, a revision of all terms and definitions was conducted by a Delphi approach in 3 steps by neurosurgeons (fetal/paediatric/adult), urologists (paediatric/adult), rehabilitation medicine specialists, fetal medicine and perinatal imaging specialists, geneticists, pathologists, nephrologists and patient representatives, all members of the International Federation for Spina Bifida and Hydrocephalus (IFSBH).

**Results:**

In the first instance, 39 experts reviewed and refined the terminology that could be used to describe the anatomical characteristics of all forms of SBoD. At the second stage, 24 experts established terms and unambiguous definitions for 16 skin findings, 7 bone findings and 33 spinal cord findings that were considered essential features capable of describing all forms of spinal dysraphism. In the third stage, 29 experts validated 24 spinal dysraphic anomalies using these pre-agreed findings. All terms and definitions were validated by vote with a threshold of 80% approval (abstention was permitted). No terms with disagreement were retained in the subsequent classification.

The revised SBoD classification was transferred to the Orphanet nomenclature (ORPHA:823). 16 existing ORPHAcodes were deemed obsolete, 10 ORPHAcodes were updated (terms and/or textual definitions) and 25 new ORPHAcodes were created. The SBoD working group also developed a ‘decision tree’ for new users, to assist them in the practical aspects of applying the revised classification and designating appropriate ORPHAcodes.

**Conclusions:**

An update of the Orphanet Classification of spinal dysraphism was conducted by a European interdisciplinary group of experts encompassing all aspects of healthcare for patients with these disorders. This new classification, based on essential skin, bone and spinal cord findings offers a more logical and reproducible means to categorise SBoD. It is hoped that this will permit more precise disease delineation, consistent diagnostic accuracy and better prognostication.

## Background

The term spinal dysraphism encompasses a range of congenital malformations arising from abnormal spinal cord development. Historically, these malformations are broadly categorized into open (“*spina bifida aperta*”) and closed (“*spina bifida occulta*”) types [1] and are associated with a wide spectrum of physical and cognitive disability. A precise and unambiguous description of the various types of spinal dysraphism is crucial to ensure diagnostic consistency, prognosis evaluation and treatment stratification. Additionally, given the multidisciplinary input required for these patients, a consistent and shared vocabulary is important across all involved healthcare professionals (neurosurgeons, rehabilitation medicine, urologists, fetal medicine and perinatologists, radiologists, geneticists).

In the last decade, new dysraphic entities have been described and various classifications of spinal dysraphism have been proposed, based on embryological mechanism [2] or clinical/surgical considerations [3]. Unfortunately, the embryological basis of dysraphism, particularly the closed dysraphic disorders is incompletely understood and so classification based on embryological perspective is largely conjectural. Additionally, the terms used to describe these disorders are variably and often inconsistently used, as a result the nomenclature has become confused. The pre-2023 releases of ORPHAcodes from the Orphanet classification of rare diseases [4] were considered outdated and to have limited clinical utility. For example, some well-known disorders such as dermal sinus and limited dorsal myeloschisis (LDM) were incompletely defined in the classification, and this nomenclature was insufficient to describe new intermediate entities such as myelic limited dorsal malformation (MyeLDM) [5, 6] moreover junctional neurulation defects [7, 8] were not covered at all. Complex, combined forms of dysraphism such as a split cord malformation associated with a neural tube closure defect were also difficult to describe using previous ORPHAcodes.

In Orphanet classification [9] a *disorder* is defined as a clinical entity characterised by a set of homogeneous phenotypic abnormalities (symptoms and signs or ‘findings’) and an evolution allowing a definitive clinical diagnosis. In contrast to disorders, *findings* are observations, judgments, or assessments of a patient *supporting* rather than *defining* the presence of a disorder. Therefore, a finding cannot exist independently from a disorder. For example, Chiari II malformation was previously listed as a disorder, however in the most recent version it is considered a finding since Chiari II malformation does not exist outside the disorder of open neural tube defect.

In an attempt to resolve the current confusion in nomenclature, two European reference networks (ERNs), namely ITHACA and eUROGEN, combined expertise in order to comprehensively revise the classification of spinal dysraphism (SD). ITHACA is an ERN for rare malformation syndromes and rare intellectual and neurodevelopmental disorders, and eUROGEN is an ERN for rare urogenital diseases and complex conditions in both children and adults. This article describes the revision process of the Orphanet SBoD classification and the proposal of a ‘tree-based method’ to navigate the revised classification.

## Methods

The Delphi methodology was used to build consensus among experts from the two participating ERNs during three in-person workshops. The Delphi sessions were performed using a voting app (Wooclap®), coordinated by FD and TSD. Iterative voting sessions were conducted anonymously by experts for all terms and definitions. The consensus thresholds applied are detailed in the following sections, separately for findings and for disorders. The terminology was edited using an ontology editor (Protégé v5.6 ®/HermIT reasoner v1.4.3). The coordination of the workshops was provided by GM and JMJ, leaders of the SBoD WG.

### Preliminary work

During the preparatory work for the revision, it was decided to avoid reference to embryological or mechanistic terms and to focus on descriptive terms only. It was agreed that all disorders under the umbrella term of SD could be morphologically described as a combination of three categories of findings (or phenotypes) namely skin findings, bone findings and spinal cord findings. This proposal was initially tested on the following types of SD: conus lipoma, dermal sinus, diastematomyelia, filum lipoma, myelomeningocele (MMC), LDM, MyeLDM and neuroenteric cyst. It was demonstrated that these malformations could be logically described, using semantic reasoning from a prescribed list of findings. A first list of terms (along with supportive images) for the description of these findings was established by a paediatric neurosurgeon and a fetal medicine expert, based on a series of patients with SD from one expert Rare Disease (RD) centre.

To reiterate, ‘findings’ refer to components (skin, bone or spinal cord) of a SD, whereas ‘disorders’ refer to discrete diagnostic entities (actual spinal dysraphisms) that are typically a combination of findings. Additionally, ‘terms’ refer to words used to describe a finding or a disorder (main term and synonyms).

These findings are compatible with the definition of malformation phenotypes: “observable traits of an organism, comprising its morphology, its physiology at the level of the cell, the organ, and the body” [10]. In the context of SD, we considered both fetal phenotypes (mainly ultrasound findings but also MRI, CT scan, amniotic fluid biology, fetal surgical observations) and postnatal phenotypes (clinical and imaging, biology, gene expression profiles when known, surgical and pathological observations).

### First round

The initial finding terms were evaluated for their clarity, lack of ambiguity and relevance to SD. All terms were presented to the expert panel by the coordinators, each was discussed, edited, and approved by vote. Then, the coordinators called for additional or missing terms from all medical specialists represented on the panel.

All terms, that at least 50% of the panel agreed were appropriate descriptors of spinal dysraphism were carried forward to the next round. The additional terms suggested by the experts were collected and curated (only finding terms were kept, and synonyms were grouped).

### Second round

During the second round the definitions of the previously agreed descriptive terms were agreed by consensus. Each finding was given a main term and textual definition, and the list was scrutinised for synonyms. Each finding was supported by representative images (photo, ultrasound, MRI, CT-scan) where possible. A threshold of 80% approval was used for validation of the terms.

The coordinators next conducted a review of existing Orphanet entities and established a list of candidate SD disorders with textual definitions based on combinations of validated skin, bone and spinal cord findings along with supportive PubMed citations. In compliance with Orphanet policies, only disorders with supportive publications referring at least 2 cases or a case series were considered.

At the end of the second round a decision-tree was established that allowed SD disorders to be identified through a sequence of questions on the presence or absence of findings.

### Third round

During the third round, a proposed hierarchical organization of SD disorders was presented to the panel of experts and the definitions of SD, based on specific combinations of findings and previously agreed terms were reviewed and revised through consensus. Electronic voting was conducted to approve and validate each term/synonym/definition for all disorders. A threshold of 80% of approval was used for validation.

The terms and definitions of SD were edited by the Orphanet scientific and editorial teams to comply with their policies, linked to existing publications (PubMed citations). Before publication within the Orphanet knowledge base, all terms and definitions underwent a final review by the SBoD coordinators and patient representatives. All SD entities were organized in a hierarchical manner, with respect to the 3 complexity levels used to organize the Orphanet classification (*group of disorders*, *disorder* and *subtype of a disorder*), as detailed in Orphanet procedures [9].

A *group of disorders* is a level that represents a collection of clinical entities sharing a set of common features. A *disorder* is a clinical entity characterized by a set of homogeneous phenotypic abnormalities (e.g. *findings*) and evolution allowing a definitive clinical diagnosis. The disorder level is the aggregation level, i.e. the level of data sharing and statistical reporting in the EU. A *subtype of a disorder* is a subdivision of a *disorder* according to a positive criterion. This structure of the Orphanet classification allows a multi-hierarchical and polyparental organisation of rare diseases.

## Results

The initial set of findings comprised 13 skin findings, 4 bone findings and 26 spinal cord findings with supporting images (pictures, US images, MRI images) from a series of 47 cases of patients with SD. The first round served to designate an initial set of findings that could be used to describe all entities of SD. In the second round, all relevant findings and their definitions were validated. The third round focused on establishing the disorders, defined as specific combinations of previously validated findings.

### First round

During the first round, 39 healthcare professionals and patient representatives participated (Table 1). There was 92% (36/39) agreement for the proposed model of a descriptive classification of SD based on predefined essential criteria (combinations of findings). Three of 39 participants (8%) abstained.

**Table 1:** Profiles of experts participating in the classification revision Delphi process

Expertise profiles	Round 1	Round 2	Round 3
Neurosurgeon (pediatric/fetal/adult)	7	8	6
Urologists (pediatric/adult)	13	3	2
Rehabilitation medicine	2	2	4
Fetal medicine/perinatal imaging	8	7	8
Geneticist	5		1
Other (pathologist, nephrologist)	2		
Patient representatives	2		4
Total	39	24	29

A total of 12 skin findings (vote range 53–83%) and 3 of the 4 bone findings (vote range 71–90%) were deemed appropriate for SD description. The term “spina bifida” failed to attain a majority vote for validation (29%). Fourteen of the 24 spinal cord findings were deemed appropriate for SD description (vote range 50–97%).

The panel proposed a total of 23, 32 and 43 additional terms for skin, bone and spinal cord findings, respectively.

At the end of the first round, consolidation of terms was achieved though grouping of synonyms under a main term, and duplicate/redundant terms resulting in a new list of findings for the second round.

### Second round

During the second round, 16 skin findings with three levels of organization were defined and approved (Table 2), 7 bone findings with two levels of organization were defined and approved (Table 3) and 33 spinal cord findings with four levels of organization were defined and approved (Table 4). Each of the terms were validated with a written definition and synonyms were acknowledged with a minimum of 80% agreement. In some instances, experts abstained from the vote when they considered the finding outside their area of expertise. In all cases, a majority (> 50%) of experts cast a vote.

**Table 2:** Skin findings associated with spinal dysraphism

Finding	Definition
S.1: no skin coverage	Skin defect sufficient for a risk of cerebrospinal fluid leak or exposure of neurologic tissue
S.1.1: neural Placode	The spinal cord is opened at the surface of the skin, loosing its tube shape to look like a plate like structure
S.1.2: incomplete skin covered sac	Saccular lesion with a thin transparent membrane bordered with skin
S.2: skin Coverage	There is complete skin all over the spine area
S.2.1: normal skin	No skin stigmata (absence of abnormal relief or color of the skin)
S.2.2: cutaneous stigmata	Presence of abnormal relief or color of the skin
S.2.2.1: caudal appendage	Lumbosacral appendage covered by skin. Synonyms: caudal appendiculum, tag, lumbar skin appendage, pseudo-tail
S.2.2.2: cutaneous angioma	Cutaneous lesion with blood vessels, with variable redness or volume (thickened forms are more likely to be associated with SD) Synonyms: cutaneous hemangioma, skin angioma, skin hemangioma
S.2.2.3: cutaneous swelling	The skin is elevated by a subcutaneous mass (can be solid or cystic), physical examination can discriminate between cystic mass (meningocele can be depressed by pressure) and solid mass (usually subcutaneous lipoma, non depressible) Synonyms: cutaneous bump
S.2.2.3.1: cystic cutaneous swelling	cystic mass covered by skin with normal thickness but possible abnormal color; physical examination: can be depressed by pressure Synonyms: skin-covered sac
S.2.2.3.2: solid cutaneous swelling	Solid mass covered by skin: soft tissue swelling beneath skin, usually subcutaneous lipoma; physical examination: non depressible (solid)
S.2.2.4: cutaneous dimple	Focal skin depression, location can be median (midline dimple) or paramedian (paramedian dimple) Synonym: skin dimple
S.2.2.5: cutaneous dyspigmentation	Hyper and/or hypopigmentation of the skin Synonym: skin dyspigmentation
S.2.2.6: cutaneous pit	Cutaneous depression with abnormal/no skin in the middle Synonyms: cutaneous hole, cutaneous crater
S.2.2.7: natal cleft deviation	Intergluteal crease is deviated and/or bifid Synonyms: gluteal fold deviation, intergluteal cleft deviation
S.2.2.8: lumbar hypertrichosis	Focal area hypertrichosis of the lumbar region Synonym: faun tail

**Table 3:** Bone findings associated with spinal dysraphism

Finding	Definition
B.1: bony septum of the spinal canal	Anteroposterior bone separation between two dural sac containing each one spinal cord from the split cord (eg. in split cord malformation type 1) Synonyms: intracanalicular bony septum; intracanalicular bony spur
B.2: split vertebra	The body of the vertebra is splitted into 2 halves (usually triangular-shaped) Synonym: butterfly vertebra
B.3: hemi vertebra	Laterally-based wedge vertebra with one single pedicle and hemi-lamina
B.4: spina bifida	Defect of posterior vertebral arch resulting in a spinous process bifidity, the, vertebral posterior arch is open, the laminae vertebra can be convergent, parallel or everted Synonyms: open posterior arch; posterior arch fusion defect
B.4.1: open and convergent posterior laminae vertebra	The posterior laminae vertebra are open and convergent
B.4.2: open and parallel posterior laminae vertebra	The posterior laminae vertebra are open and parallel
B.4.3: open and everted posterior laminae vertebra	The posterior laminae vertebra are open and everted

**Table 4:** Spinal cord findings associated with spinal dysraphism

Finding	Definition
SC.1: normal-shape conus	The spinal cord tapers to a single point
SC.2: abnormal-shape conus	The morphology of the termination of the spinal cord is abnormal Synonym: abnormally shaped conus
SC.2.1: open conus	The termination of the spinal cord is splayed (eg. in case of terminal myelocystocele or myelomeningocele) Synonym: trumpet conus
SC.2.2: attenuated conus	The termination of the spinal cord is elongated and poorly defined Synonyms: asymptotic conus, ill-defined conus
SC.2.3: truncated conus	The termination of the spinal cord is blunted or club-shaped (as observed in case of caudal regression)
SC.3: double dural sac	Two thecal sacs, each containing an hemi-cord, usually seperated by an osseo-cartilageneous septum (eg. in case with split cord type 1)
SC.4: intradural septum	Septum occurring within a single dural tube, separating two hemi-cord (as observed in cases of split cord malformation type 2)
SC.5: fatty filum	The *filum terminale* contains fat tissue
SC.6: intradural inclusion cyst	Intrathecal solid/cystic mass comprising dermal or epidermal elements Synonyms: intradural epidermoid cyst, intradural dermoid cyst
SC.7: low lying cord	The end of spinal cord is observed below the lower level of L2 vertebra Synonym: descent of medulla
SC.8: spinal meningocele	Herniation of the thecal sac outside of the spinal canal
SC.8.1: anterior spinal meningocele	Herniation of the thecal sac through a defect in the anterior spinal elements
SC.8.2: posterior spinal meningocele	Herniation of the thecal sac through a defect in the posterior spinal elements
SC.9: neural bud	Focal attachment of the terminal neural elements to the dome of a sac
SC.10: spinal cord lipoma	Adipose mass contiguous within or attached to the spinal cord
SC.10.1: intramedullary spinal cord lipoma	The lipoma is confined within the spinal cord (The pia is intact)
SC.10.2: extramedullary spinal cord lipoma	The lipoma extends beyond the confine of the spinal chord (The pia is not intact)
SC.10.2.1: conus region lipoma	Spinal lipoma all or part of which has attachment to the conus Synonym: spinal cord conus lipoma
SC.10.2.1.1: posterior conus region lipoma	The distal part of the conus is distinct from the lipoma Synonym: dorsal spinal cord conus lipoma
SC.10.2.1.2: terminal spinal cord lipoma	The lipoma appears as a caudal extension of the termination of the spinal cord Synonym: terminal spinal cord conus lipoma
SC.10.2.1.3: transitional spinal cord conus lipoma	The lipoma appears as a posterior extension of the termination of the spinal cord Synonym: dorso-terminal lipoma, transitional spinal cord lipoma
SC.10.2.2: dorsal spinal cord lipoma	The lipoma is attached to the posterior aspect of the spinal cord and is above the conus
SC.10.2.3: chaotic spinal cord lipoma	Part of the lipoma extends ventral to the DREZ (dorsal root entry zone)
SC.10.2.4: extra-canalar spinal cord lipoma	The interface between the fat tissue and the spinal cord is (wholly or partly) outside the spinal canal
SC.10.2.5: lipoma with rotated placode	The interface between the lipoma and neural tissue is rotated Synonym: rotated placode
SC.10.2.6: transdural lipoma	The lipoma extends through a defect in the dura
SC.11: stalk	Persisting connection between the skin and the spinal cord Synonym: sinus track, sinus tract
SC.11.1: dermo-epidermal stalk	The stalk is composed of skin epithelium Synonym: dermal sinus track
SC.11.2: fibroneural stal	The stalk is composed of fibrous and neuroglial tissue Synonym: LDM track
SC.11.3: lipomatous stalk	The stalk is composed of adipose tissue
SC.12: split cord	Division (clefting) of the spinal cord Synonym: diplomyelia, diastematomyelia
SC.13: myelocystocele	Cystic dilatation of the central canal of the spinal cord, herniating posteriorly through a spinal defect. A part of the spinal cord is outside the spinal canal
SC.14: extra-canalar spinal cord	A part of the spinal cord is outside the spinal canal

All skin findings were clinically visible signs (Table 2). The dichotomy between “open” and “closed” dysraphisms was preserved in the description of skin findings. Some prenatal imaging findings (e.g. Chiari II malformation or small posterior fossa) were considered to be indirect evidence of a distal open neural tube defect with incomplete skin coverage and thus were considered as skin finding proxies during prenatal evaluation [11]. Cutaneous swelling, denoting a protuberant mass over the midline spine was retained as a useful and relevant descriptor in keeping with the historical Tortori-Donati classification [1].

A limited number of osseous findings were validated (Table 3), reflecting the small number of vertebral anomalies found in association with SD (most commonly split spinal cord or open posterior arch). The term “spina bifida” was included in the category of bone findings to denote open posterior laminae, which could be subclassified as convergent, parallel or everted.

Spinal cord findings (Table 4) were mostly imaging based (MRI, ultrasound), however some could be confirmed only by subsequent histopathology (e.g. subcategories of neurocutaneous stalks) or observed during surgery (e.g. chaotic lipoma).

Low lying cord was defined as a spinal cord termination below the lower level of the L2 vertebra, and not as a synonym of the term ‘tethered cord’, which, the panel agreed should refer to a set of clinical symptoms rather than a discrete pathological entity.

Extrusion of the spinal cord or neural placode was validated as a finding, as there is evidence that this may impact on prognosis by imparting additional mechanical stress (traction) applied on the spinal neural structures (spinal cord and nerve roots).

### Third round

Using the validated findings, a decision tree was constructed in which the findings could be combined to define a particular SD disorder. In this way a decision tree a total of 24 SD disorders were described, each with an agreed (minimum of 80% agreement of the panel) textual definition and list of synonyms. Experts abstained from voting when they considered the finding outside their area of expertise, nonetheless in all cases a majority (> 50%) of the panel voted for each disorder.

Finally, in conjunction with the Orphanet team the new classification was incorporated into the Orphanet nomenclature of Rare Diseases.

### Transfer of the SBoD classification to Orphanet

The 24 expert-validated SD disorders were discussed and affirmed by the Orphanet Medical and Scientific Committee, to ensure compliance with Orphanet guidelines and consistency across the entire Orphanet classification.

The 24 entities were integrated into Orphanet nomenclature at the level of disorder (n = 18) or at the level of subtypes of disorders (n = 6). Additionally, integration of the new classification of SBoD necessitated the creation of groups of disorders to build the hierarchy: 6 new groups were created, and 5 existing groups were updated.

As a result of the update of the Orphanet SBoD classification, 16 previous ORPHAcodes were deemed obsolete, 10 ORPHA codes were updated (terms and/or textual definitions) and 25 new ORPHAcodes were created. The updates included terminological updates (terms and synonyms), textual definition updates and updates at the level of classification (group, disorder and subtype). All disorders were organized under the group named “Spina bifida and other spinal dysraphisms” (https://www.ORPHA:823).

The 10 updated Orphanet entities and the 25 created Orphanet entities are listed in Table 5 and Table 6 respectively. The 16 Orphanet entities that were deemed obsolete comprise: Arnold Chiari malformation type 2, Total spina bifida aperta, Thoracolumbosacral spina bifida aperta, Lumbosacral spina bifida aperta, Cervical spina bifida aperta, Cervicothoracic spina bifida aperta, Upper thoracic spina bifida aperta, Total spina bifida cystica, Thoracolumbosacral spina bifida cystica, Lumbosacral spina bifida cystica, Cervical spina bifida cystica, Cervicothoracic spina bifida cystica, Upper thoracic spina bifida cystica, Lipoma associated with neurospinal dysraphism, Leptomyelolipoma, and Lipomyelomeningocele.

**Table 5:** Spinal dysraphism terms updated in the revised classification

Level	New terms (updates)
Group of disorders (*n* = 5)	Spina bifida and other spinal dysraphisms (ORPHA:823) Open spinal dysraphism (ORPHA:268369) Spinal dysraphism with posterior meningocele (ORPHA:268744) Split cord malformation (ORPHA:573278) Myelocystocele (ORPHA:268813)
Disorder (*n* = 4)	Split cord malformation type 1 (ORPHA:1671) Split cord malformation type 2 (ORPHA:573253) Isolated posterior meningocele (ORPHA:268810) Caudal regression syndrome (ORPHA:3027)
Disorder subtype (*n* = 1)	True Myelomeningocele (ORPHA:645383)

**Table 6:** Spinal dysraphism terms created in the revised classification

Level	New terms (creations)
Group of disorders (*n* = 6)	Open spinal dysraphism with a posterior meningocele (ORPHA:645270) Closed spinal dysraphism (ORPHA:645202) Conus spinal cord lipoma (ORPHA:645367) Dysraphism with stalk (ORPHA:645193) Limited dorsal myeloschisis (ORPHA:645196) Fibrolipomatous filum anomaly (ORPHA:645279)
Disorder (*n* = 14)	Myeloschisis (ORPHA:645398) Open spinal dysraphism with a myelomeningocele (ORPHA:93969) Myelic limited dorsal malformation (ORPHA:645378) Dorsal spinal cord lipoma (ORPHA:645362) Intramedullary non-dysraphic spinal cord lipoma (ORPHA:645359) Saccular spinal dysraphism with a stalk to the dome (ORPHA:645319) Saccular limited dorsal myeloschisis (ORPHA:645354) Non-saccular limited dorsal myeloschisis (ORPHA:645343) Non-terminal myelocystocele (ORPHA:645340) Terminal myelocystocele (ORPHA:645337) Spinal dermal sinus (ORPHA:645188) Retained medullary cord (ORPHA:645334) Isolated filum lipoma (ORPHA:645325) Isolated transitional filum lipoma (ORPHA:645322)
Disorder subtype (*n* = 5)	True myeloschisis (ORPHA:645401) Hemi-myeloschisis (ORPHA:645393) Hemi-myelomeningocele (ORPHA:645388) Fibroneural non-saccular limited dorsal myeloschisis (ORPHA:645310) Lipomatous non-saccular limited dorsal myeloschisis (ORPHA:645300)

## Discussion

### Strength and limitations

For more than two decades new SD entities and classifications have been advocated, this reflects the inadequacy of previous classifications to accommodate new dysraphic entities [1, 12, 13]. Previous attempts to revise SD classification are typically based upon expert opinion of a single author or small groups of authors, mostly neurosurgeons, and are commonly based upon hypothetical rather than confirmed embryo-pathological mechanisms.

In this international collaboration, we have attempted to achieve inter-disciplinary consensus in developing a classification that is descriptive, reproducible and reflects the range of SD disorders recognised in contemporary practice.

Previous SD classifications are based on a compartmental structure and therefore have an inherent inflexibility in which to describe the spectrum of dysraphic anomalies encountered in everyday practice. By producing a large thesaurus of defined findings, capable of being combined to describe existing as well as new categories of disorders, we hope to have achieved a robust SD classification which can be used across disciplines and that can also evolve over time. By avoiding reference to embryological mechanisms, and by establishing an agreed vocabulary by which malformations can be logically described we anticipate that, unlike previous classifications, this will be durable, unambiguous and will be able to incorporate any future SD entities that might be described.

To our knowledge this is the first large SD classification able to encompass prenatal diagnoses—a requirement that has become increasingly necessary in the era of fetal surgery for open dysraphism. The classification permits a common interdisciplinary language to describe these disorders and will also and allow for prenatal to postnatal follow-up and correlations.

The multiple levels in the revised classification (group of disorders, disorder, subtype of disorder) provide a solution to cover all SD entities, thus avoiding any ‘Unclassified Dysraphic Observations’ (UDO). Thus far, during informal testing on various sample SD cases we have not encountered any ‘UDO’ cases that cannot be described using this new classification.

The methods used to design this revised classification comply with the requirements expected in standard healthcare terminologies [4]. Consequently, interoperability offered by this classification is an opportunity to become a pivot tool for SD descriptions. It is hoped that this approach and classification offers a readily useable tool not only for individual cases but also for the description of patient cohorts within the context of clinical studies and public health surveillance. Disseminating and implementing this classification will require education of professionals and the working group intend to provide tutorials, and develop an App based tool to facilitate this.

### Decision factors for terms and organisation

**Fig. 1:**
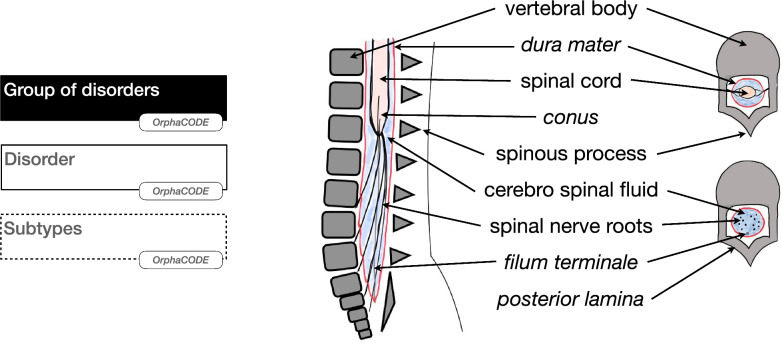
Classification legends for Orphanet entities representation (left) and for normal anatomy representations (right)

An overriding principle used in the development of the classification was that all salient SD findings should refer to factors of potential discriminating and prognostic significance. These can be considered under four headings:

(i) The relationship of the neural tissue to the surrounding skin e.g. open or closed.
(ii) The morphological status of the spinal cord and roots, specifically divergence from normal anatomy indicating inherent dysplasia or an irreversible malformative state. (differences from normal anatomy described in Fig. 1)
(iii) Evidence of mechanical distortion on neural structures through compression or traction.
(iv) Evidence of an adverse environment for the spinal cord.

**Fig. 2:**
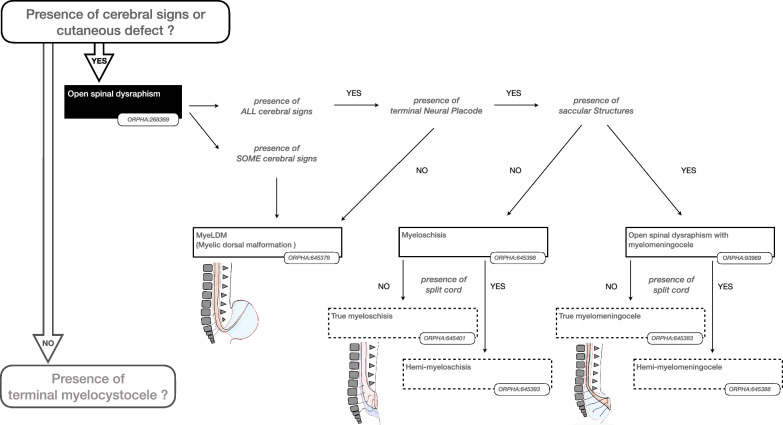
Navigation in the Spina Bifida and other Dysraphism (SBoD) classification: The open spinal dysraphism disorders

**Fig. 3:**
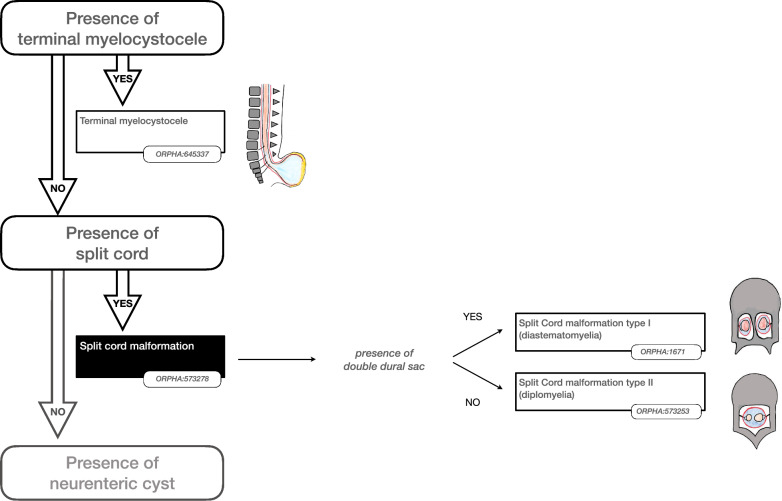
Navigation in the Spina Bifida and other Dysraphism (SBoD) classification: The closed spinal dysraphism disorders (1/4)

**Fig. 4:**
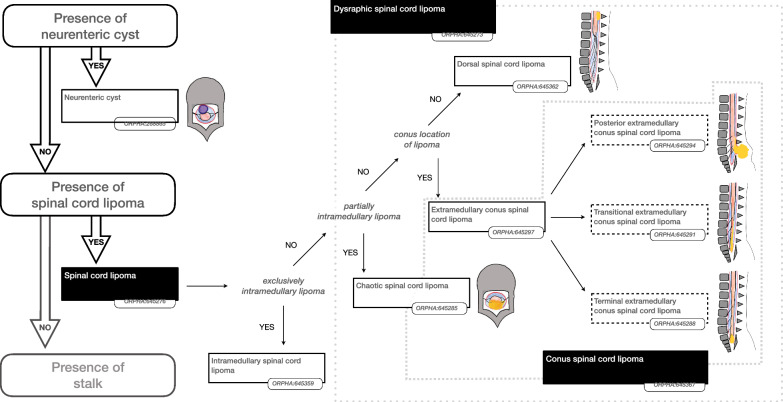
Navigation in the Spina Bifida and other Dysraphism (SBoD) classification: The closed spinal dysraphism disorders (2/4)

**Fig. 5:**
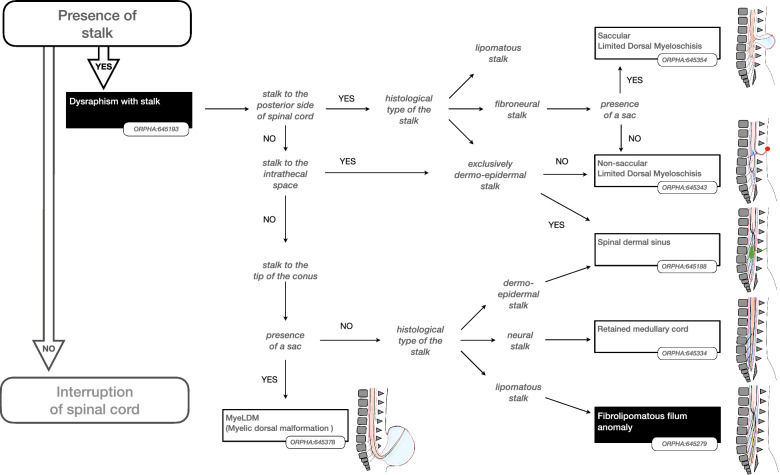
Navigation in the Spina Bifida and other Dysraphism (SBoD) classification: The closed spinal dysraphism disorders (3/4)

**Fig. 6:**
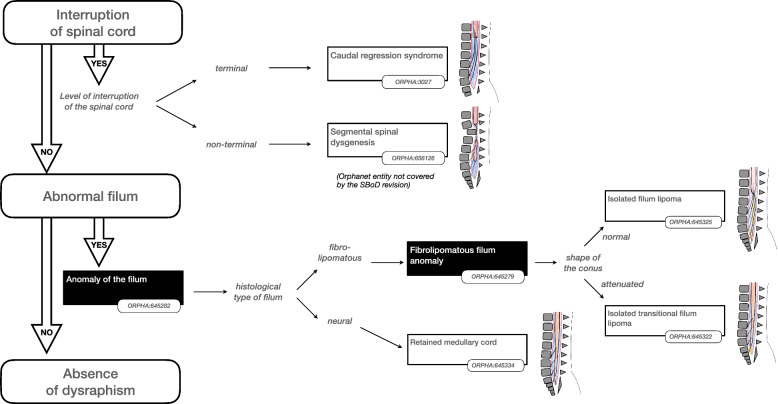
Navigation in the Spina Bifida and other Dysraphism (SBoD) classification: The closed spinal dysraphism disorders (4/4)

Some findings were considered important to prioritise the hierarchy of disorders. The presence or absence of such “major findings” underpinned the reasoning to organise the classification as shown in the Figs. 2, 3, 4, 5 and 6. More precisely, these findings are either present or absent, thus avoiding the risk of overlapping categories of SD disorders: open (Fig. 2)/split cord (Fig. 3)/spinal cord lipoma (Fig. 4)/stalk (Fig. 5)/filum anomaly (Fig. 6).

When consensus on a term was unmet, we ensured that the corresponding SD could be incorporated under another agreed entity (e.g.: Lipomyelomeningocele, was deemed obsolete but newly designated under Conus spinal cord lipoma, ORPHA:645367). The vertebral level of the malformation was not included in the classification as it was considered this would add unnecessary complexity, and whilst the level of the lesion might have prognostic significance, it is not a factor specific to a particular type of SD.

### Evolution of the “open versus closed” paradigm for SBoD

Open and closed SD disorders have been separated to emphasize important differences with respect to management and prognosis as shown in Fig. 2. Open SD finding refers to a defect of skin coverage with actual, or the potential for CSF leakage. However, this traditional dichotomy between open and closed does not accurately reflect the spectrum of dysraphisms in terms of skin coverage and diverse consequences on the brain.

Non-terminal myelocystocele (ORPHA:645340) is a relatively recently described entity and has assumed particular importance in prenatal diagnosis since it can easily be misdiagnosed as myelomeningocele but has a much more favourable prognosis. From an imaging and prognostic perspective, non-terminal myelocystocele can be considered as a closed SD yet the associated sac may have a poor or incompletely epithelialized covering with the risk of CSF leakage requiring early surgical closure. However, non-terminal myelocystocele is not associated with the intracranial features of Chiari II complex and therefore it is classified as a closed SD because of the normal brain findings.

### Frontier forms of dysraphisms

One of the objectives of the SBoD WG was to incorporate newly described dysraphic entities that postdate existing classifications. This includes ‘intermediate’ forms of dysraphism that are difficult to fit into existing discrete categories. Examples include “junctional neurulation” disorders that are thought to be due to maldevelopment at the interface between primary and secondary neurulation [8] and “MyeLDM” (ORPHA:645378) a term created by contraction of “Myelomeningocele” and “LDM”. MyeLDM is characterised by a CSF containing sac (or “*coel”*) reminiscent of myelomeningocele (ORPHA:93969), but this is skin covered akin to the saccular form of LDM (ORPHA:645354). Like non-terminal myelocystocele (ORPHA:645340) the distinction has become of particular relevance in prenatal diagnosis and selection of cases for prenatal surgery.

### Conus region lipomas

The new classification recognises variations within two forms of conus region lipoma: firstly, the transitional extramedullary conus spinal cord lipoma (ORPHA:645291) may comprise lipoma on the dorsal aspect of the conus and is referred to as posterior extramedullary conus spinal cord lipoma (ORPHA:645294), or the lipoma may continue distally from the conus and is referred to as terminal extramedullary conus spinal cord lipoma (ORPHA:645288) (Fig. 4). Secondly, the Isolated transitional filum lipoma (ORPHA:645322) may share characteristics between the isolated filum lipoma (ORPHA:645325) and the terminal extramedullary conus spinal cord lipoma (ORPHA:645288) (Fig. 6).

### Myelomeningocele and myeloschisis

These two entities represent the two main forms of open dysraphism (Fig. 2). They have in common a dysplastic termination of the spinal cord (terminal placode) but differ in that the placode is elevated outside the spinal canal by a sac (myelomeningocele) or remains at the level of the spinal canal (myeloscisis). The distinction has been found to be of prognostic significance for fetuses undergoing prenatal closure [14].

### Split cord malformations

The distinction between split cord malformations (SCMs) type I and type II has been retained [15] (Fig. 3). There was consensus that the distinguishing feature between the two SCM types was the presence of a double or single dural sac.

In a single case report, a bony spur within a single sac has been reported but with inconclusive illustration and documentation [16]. Consequently, we did not create a specific category of SCM to cover this entity. Therefore a mesenchymatous/fibrous septum within a single sac was considered pathognomonic of SCM type 2. The term “diastematomyelia” has been kept as a synonym for cases with an intermedullary bony spur, i.e. SCM type 1, similarly “diplomyelia” is retained as a synonym referring to a SCM type 2.

SCMs are postulated to arise at an early stage of embryological impairment (gastrulation), and this can initiate a cascade of events resulting in additional malformations including various types of filum anomalies, spinal cord lipoma and stalk. In case of open dysraphism associated with a split cord the impact of the open component mandates that these are considered open subtypes of SD, for example Hemi-myelomeningocele (ORPHA:645388).

### Lipomatous malformations

Spinal lipomas are characterised by adipose tissue within, or attached to, the spinal cord. Most commonly these are attached, or adjacent to the conus medullaris. It is important to distinguish lipomas of the filum from those of the conus as it has long been recognised that the latter are more likely to have dysplasia of the terminal spinal cord and a less favourable prognosis [14].

Filum lipoma is therefore classified under anomaly of the filum (ORPHA:645282) and this diagnosis excludes contiguity between spinal cord and the lipoma.

Intramedullary spinal cord lipoma (ORPHA:645359) is included in the classification for completion although it is not generally considered to be a dysraphic anomaly (Fig. 4).

The term “lipomyelocystocele” is retained in the Orphanet classification as a keyword pertaining to the wider group of myelocystocele (ORPHA:268813), it is not considered to be a distinct disorder or a subtype of lipoma. Myelocystocele generally carries a poor functional prognosis primarily due to the dysplastic, dilatated termination of the spinal cord; a lipomatous component may be present but this is considered to be an epiphenomenon.

After explicit definitions of the lipomatous malformations above, dysraphic spinal cord lipomas (ORPHA:645273) were defined as a discrete group of disorders in which the lipoma is described as”extramedullary” in contrast to Non-dysraphic intraspinal lipoma (ORPHA:645359) [17]. Hitherto, the location and degree of dysplasia have been the major criteria for the categorization and description of dysraphic spinal cord lipomas [12, 18]. The current classification maintains much of the existing nomenclature but excludes any reference to embryopahthogenesis. The following dysraphic lipomatous malformations are now recognised:

- Chaotic spinal cord lipomas; characterised by the presence of lipoma beyond the DREZ, a finding that may only be determined a surgery. This indicates significant dysplasia of the conus and portends a worse prognosis [18].
- Dorsal spinal cord lipoma (ORPHA:645362) is reserved for dysraphic lipomas on the dorsal aspect of the spinal cord that *do not* involve the conus and therefore excludes conus lipomas.
- Conus region lipoma (ORPHA:645297) comprise all lipomas that completely or partially involve the conus and are subgrouped as caudal or transitional in accordance with the existing Chapman classification [19]. The current classification includes additional descriptive terms such as transdural lipoma, extracanalar lipoma or rotated placode. These terms allow for further refinement of the classification of lipomas, however more clinical data is required to ascertain whether these features have prognostic significance.

### Dysraphism with Stalk

This category refers to the entity of Limited Dorsal Myeloschisis (LDM) [5]. The SBoD group recognised that there was considerable variation in the terminology to describe this subset of dysraphic anomalies (dermal sinus track, short and long tethering track, meningocele manque etc.). In an attempt to improve diagnostic precision the current classification identifies specific exclusion criteria but permits inclusion of histopathological findings (Fig. 5). For example, a diagnosis of LDM implies the absence of spinal cord lipoma and presence of a single spinal cord. However in recognition of the continuity between the spinal dermal sinus (ORPHA:645188) and the non-saccular LDM (ORPHA:645343) [20], and the increased infection risk associated with the former, the new classification permits the refining the diagnosis once the histological nature of the neurocutaneous stalk has been established.

A SD with stalk can be observed prenatally especially when associated with a posterior meningocele. The spinal cord origin of the stalk impacts on prognosis and therefore a distinction is made between a stalk originating on the posterior surface of the spinal cord (as defined by Pang for LDM [5]) and a stalk at the tip of the (potentially dysplastic) conus referred to as MyeLDM (Fig. 5). Both disorders are part of the group saccular spinal dysraphism with a stalk to the dome (ORPHA:645319).

### Interrupted spinal cord

The existing disorder caudal regression syndrome (ORPHA:3027) includes an abnormality of the terminal spinal cord but has additional gastrointestinal, genitourinary, skeletal and neurological manifestations. The working group included the finding of truncated conus to describe the essential spinal cord anomaly associated with this syndrome (Fig. 6). The recently debated entities of spinal segmental dysgenesis and junctional neural tube defect were not covered by the SBoD WG.

### Anomaly of the filum

Anomalies of the filum (ORPHA:645282) are a group of disorders organized according to the composition of the *filum terminale* (fibrolipomatous, neural, etc.) as well as the shape of the conus at the point of attachment (Fig. 6).

Isolated filum lipoma (ORPHA:645325) comprises fatty thickening of the filum attached to a normally shaped conus regardless of spinal level whilst retained medullary cord (ORPHA:645334) indicates the additional presence of neural tissue within the filum and an abnormally shaped conus [21].

### Posterior sac

**Fig. 7:**
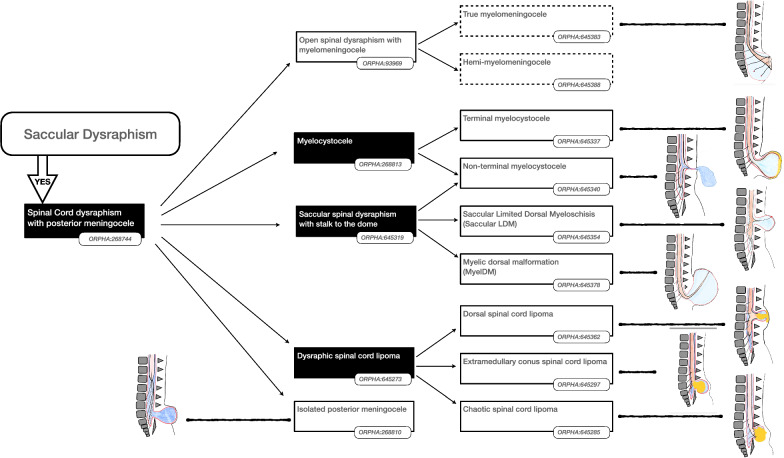
Navigation in the Spina Bifida and other Dysraphism (SBoD) classification: The saccular spinal dysraphism disorders

The term “posterior meningocele” is not included in the current classification as it was deemed insufficient to describe a single disorder or subtype. A posterior saccular finding, particularly in the prenatal setting may be seen in the context of myelomeningocele, LDM or non-terminal myelocystocele. Additional findings based on the skin coverage, the presence and position of a stalk and the existence of spinal cord lipoma are required to ascribe a specific diagnosis within the current classification (Fig. 7).

### Limitations and perspectives

This in-depth revision of the classification of SD is based on opinions from a multidisciplinary panel of European experts. In the absence of genetic markers, high quality clinical data or evidence from experimental animal models, the classification is pragmatic based on opinion and experience and recognises that some rare dysraphic malformations do not fit into current discrete diagnostic categories.

The new classification will need to be evaluated for its applicability to clinical coding, with particular attention to its utility, reproducibility and generalizability across diverse clinical settings. The development of standardized protocols, such as the recent proposal by the SBoD group for open forms of fetal dysraphism [22], is essential for ensuring consistent data interpretation and consolidating practices across centers. Additionally, the use of the new codes from this classification can facilitate precise reporting of prenatal cases and postnatal outcomes [23], enabling meaningful comparisons between case series.

## Conclusions

The revised SBoD classification is the result of an international, multidisciplinary collaboration between experts in all aspects of dysraphism care and at all ages of life. The process has been conducted using formal consensus methodology and knowledge representations. This pragmatic and descriptive classification avoids reliance on unproven embryological hypothesis, and offers a precise, consistent and unambiguous vocabulary, and therefore a more accurate method for patient coding.

We anticipate that this new classification will promote international data sharing, support robust meta-analyses, strengthen disease registries, and improve coordination of clinical care and research collaboration. By enabling the inclusion of better-defined and more homogeneous patient cohorts in future studies, it may also enhance our understanding of disease natural history and prognosis, thereby supporting more efficiently patient counselling.

## Data Availability

The updated classification of SBoD is available on the Orphanet website: https://www.orpha.net/en/disease/detail/823

## Abbreviations

**DREZ**: Dorsal Root Entry Zone
**ERN**: European Reference Network
**HPO**: Human Phenotype Ontology
**LDM**: Limited Dorsal Myeloschisis
**MMC**: Myelomeningocele
**MyeLDM**: Myelic Limited Dorsal Malformation
**SBoD**: Spina Bifida and other Spinal Dysraphisms
**SD**: Spinal Dysraphism
**RD**: Rare Disease
**WG **: Working Group

## References

[1] Tortori-Donati P, Rossi A, Cama A. Spinal dysraphism: a review of neuroradiological features with embryological correlations and proposal for a new classification. Neuroradiology. 2000;42(7):471–91. 10952179 10.1007/s002340000325

[2] Thompson DNP. Spinal dysraphic anomalies; classification, presentation and management. Paediatr Child Health. 2014;24(10):431–8.

[3] McComb JG. A practical clinical classification of spinal neural tube defects. Childs Nerv Syst. 2015;31(10):1641–57. 26351218 10.1007/s00381-015-2845-9

[4] Rath A, Olry A, Dhombres F, Brandt MM, Urbero B, Ayme S. Representation of rare diseases in health information systems: the Orphanet approach to serve a wide range of end users. Hum Mutat. 2012;33(5):803–8. 22422702 10.1002/humu.22078

[5] Pang D, Zovickian J, Wong ST, Hou YJ, Moes GS. Limited dorsal myeloschisis: a not-so-rare form of primary neurulation defect. Childs Nerv Syst. 2013;29(9):1459–84. 24013319 10.1007/s00381-013-2189-2

[6] Zerah M, de Saint DT, Garel C, Jouannic JM, Di Rocco F. An intermediate form of dysraphism: the MyeLDM. Childs Nerv Syst. 2020;36(7):1333–4. 32346789 10.1007/s00381-020-04631-5

[7] Kim KH, Lee JY. Junctional neurulation: a junction between primary and secondary neural tubes. J Korean Neurosurg Soc. 2021;64(3):374–9. 33906341 10.3340/jkns.2021.0021 PMC8128517

[8] Yang J, Lee JY, Kim KH, Yang HJ, Wang KC. Disorders of secondary neurulation: suggestion of a new classification according to pathoembryogenesis. Adv Tech Stand Neurosurg. 2022;45:285–315. 35976454 10.1007/978-3-030-99166-1_9

[9] Orphanet. Procedural document (version 05). Orphanet nomenclature and classification of rare diseases. 2023: Document URL: https://www.orpha.net/orphacom/cahiers/docs/GB/eproc_disease_inventory_R1_Nom_Dis_EP_05.pdf.

[10] Robinson PN. Deep phenotyping for precision medicine. Hum Mutat. 2012;33(5):777–80. 22504886 10.1002/humu.22080

[11] Vande Perre S, Guilbaud L, de Saint-Denis T, Maurice P, Lallemant-Dudek P, Maisonneuve E, et al. The myelic limited dorsal malformation: prenatal ultrasonographic characteristics of an intermediate form of dysraphism. Fetal Diagn Ther. 2021;48(9):690–700. 34814137 10.1159/000519060

[12] Morota N, Ihara S, Ogiwara H. New classification of spinal lipomas based on embryonic stage. J Neurosurg Pediatr. 2017;19(4):428–39. 28128702 10.3171/2016.10.PEDS16247

[13] Balani A, Chatur C, Biswas A, Oztekin O, Mankad K. Spinal dysraphisms: highlighting discrepancies in the current literature and emphasizing on the need for a consensus. Quant Imaging Med Surg. 2020;10(3):549–53. 32269916 10.21037/qims.2020.02.04 PMC7136728

[14] Muller JM, Corral Sereno E, Jimenez A, Zapata R, Echeverria S, Jara JP, et al (2024) Differences between Myeloschisis and Myelomeningocele in patients undergoing prenatal repair of Open Spina Bifida. Fetal Diagn Ther 10.1159/000538099 PMC11981588 38471477

[15] Pang D, Dias MS, Ahab-Barmada M. Split cord malformation: Part I: A unified theory of embryogenesis for double spinal cord malformations. Neurosurgery. 1992;31(3):451–80. 1407428 10.1227/00006123-199209000-00010

[16] Singh PK, Khandelwal A, Singh A, Ailawadhi P, Gupta D, Mahapatra AK. Long-segment type 1 split cord malformation with two-level split cord malformation and a single dural sac at the lower split. Pediatr Neurosurg. 2011;47(3):227–9. 22213778 10.1159/000334278

[17] Pierre-Kahn A, Zerah M, Renier D, Cinalli G, Sainte-Rose C, Lellouch-Tubiana A, et al. Congenital lumbosacral lipomas. Childs Nerv Syst. 1997;13(6):298–334. 9272285 10.1007/s003810050090

[18] Pang D. Surgical management of complex spinal cord lipomas: a new perspective. J Korean Neurosurg Soc. 2020;63(3):279–313. 32392666 10.3340/jkns.2020.0024 PMC7218203

[19] Chapman PH. Congenital intraspinal lipomas: anatomic considerations and surgical treatment. Childs Brain. 1982;9(1):37–47. 7060411

[20] Lee JY, Park SH, Chong S, Phi JH, Kim SK, Cho BK, et al. Congenital dermal sinus and limited dorsal myeloschisis: “Spectrum Disorders” of Incomplete dysjuction between cutaneous and neural ectoderms. Neurosurgery. 2019;84(2):428–34. 29618070 10.1093/neuros/nyy058

[21] Pang D, Zovickian J, Moes GS. Retained medullary cord in humans: late arrest of secondary neurulation. Neurosurgery. 2011;68(6):1500–19. 21336222 10.1227/NEU.0b013e31820ee282

[22] Guilbaud L, Carreras E, Garel C, Maiz N, Dhombres F, Deprest J, et al. Proposal for standardized prenatal assessment of fetal open dysraphisms by the European reference network for Intellectual disability, TeleHealth, Autism and Congenital Anomalies (ITHACA) and eUROGEN. Prenat Diagn. 2024;44(9):1073–87. 38898590 10.1002/pd.6618

[23] Dugas A, Guilbaud L, de Saint-Denis T, Lallemant-Dudek P, Simonnet H, Vande Perre S, et al. Outcome of children with prenatally diagnosed saccular limited dorsal myeloschisis: the importance of accurate diagnosis. Prenat Diagn. 2025;45(5):668–75. 40237726 10.1002/pd.6800 PMC12054388

[24] Dhombres F, Morgan P, Chaudhari BP, Filges I, Sparks TN, Lapunzina P, et al. Prenatal phenotyping: a community effort to enhance the Human Phenotype Ontology. Am J Med Genet C Semin Med Genet. 2022;190(2):231–42. 35872606 10.1002/ajmg.c.31989 PMC9588534

[25] Gargano MA, Matentzoglu N, Coleman B, Addo-Lartey EB, Anagnostopoulos AV, Anderton J, et al. The human phenotype ontology in 2024: phenotypes around the world. Nucleic Acids Res. 2024;52(D1):D1333–46. 37953324 10.1093/nar/gkad1005 PMC10767975

